# Microclimates growing and watering volumes influences the physiological traits of chili pepper cultivars in combating abiotic stress

**DOI:** 10.1038/s41598-025-87262-7

**Published:** 2025-02-04

**Authors:** Farhan Ahmad, Kusumiyati Kusumiyati, Mochamad Arief Soleh, Muhammad Rabnawaz Khan, Ristina Siti Sundari

**Affiliations:** 1 Department of Agronomy, Agricultural Faculty, Universitas Padjadjaran, Jl. Bandung-Sumedang km 21 Jatinangor, Sumedang, West Java Indonesia; 2 Department of Agronomy, Faculty of Crop Production Sciences, The University of Agriculture Peshawar, Peshawar, Khyber Pakhtunkhwa Pakistan; 3 Department of Agribusiness, Faculty of Agriculture, Universitas Perjuangan, Jl. PETA No. 177, Tasikmalaya, West Java Indonesia

**Keywords:** Microclimates, Physiological stress response, Abiotic stress mitigation, Climate resilience, Resource efficient, Biological techniques, Plant sciences

## Abstract

Chili peppers are a staple food for countries worldwide and are loaded with vitamins and antioxidants. One of the world’s largest chili consumers, Indonesia faces climate adversities and cash-crop pest infestations that affect its horticulture market. The present research explores microclimatic and watering for physiological performances in different chili cultivars, useful in suggesting the strategies of cultivation with a climate-resilient perception. The research was done in the Bale Tatanen, Padjadjaran University, using a Factorial Randomized Complete Block Design to analyze chili plant physiology. According to statistical analyses, cultivars did not significantly affect absolute growth rate (AGR), but growing microclimates and watering volumes did significantly affect AGR and water use efficiency (WUE). The rain shelter and screen house had the highest WUE and AGR values. Growing microclimates and cultivars significantly affected transpiration rate, stomatal conductance to water vapor and total conductance to CO_2_, with the screen house exhibiting the highest values. All three factors significantly affected the photosynthetic rate, with the greenhouse showing the highest rate. The photosynthetic photon flux density (PPFD) was likewise highest in the greenhouse. This study aimed to systematically assess these factors and it tried suggesting practices that might assist in combating the effects of abiotic stress on chili production, for its sustainability. The findings of this research would help in conceptualizing the most efficient microclimate and watering volume for chili cultivation particularly, when considering climate change challenges as well; these results could also be applied to develop guidelines which might serve helpful at resource-poor farming.

## Introduction

Spicy, colourful, and health-promoting chili peppers are integral to many cuisines worldwide. The medicinal effects of these foods are attributed to their abundance of vitamins A and C, capsaicin, and other antioxidants^1^. One of Indonesia’s essential commodities is chili; the country has consumed the most chili over the past five years, which suggests a significant demand for this product^2^. Extreme temperatures, water scarcity, humidity fluctuations, nutritional shortages, gusts of wind, diseases, and insect pest occurrences are some challenges for horticultural crops^3^. Abiotic factors such as severe temperatures, and inconsistent growing conditions pose several challenges to cultivating chilies, impacting chili peppers’ quality and physiology^4^. Stressful conditions can affect a plant’s overall health and productivity by causing stomatal closure, decreased chlorophyll content, and modifications to hormone levels^5^, leading to variations in transpiration, photosynthesis, and water usage efficiency in chili plants^6^. These changes culminate in morphological, physiological and biochemical impacts such as lower production or dry matter, altered assimilate allocation, lowered hydraulic diameter of root xylem and decreased plant growth rate^7^. Plant growth depends on photosynthesis, which is influenced by environmental factors. The CO_2_that enters the leaf through the stomata is what drives photosynthesis. Stomata are modified leaf epidermal cells that resemble two guard cells and can open holes to allow gases and water vapor to pass between the inside of the stomata and the surrounding environment^8^. Drought directly affects photosynthesis, one of the primary metabolic processes affecting crop productivity. It has been demonstrated that stomata are primarily responsible for regulating the reduction in net CO_2_ uptake via lowering leaf internal CO_2_concentrations in mild water deficit conditions^9^. Reduced pore apertures from stomata can prevent excessive water loss, but this can also restrict CO_2_entry and slow down photosynthetic activity^10^.

Chilli plants cultivated in controlled environments typically exhibit better rates of photosynthesis and assimilation than the cultivated in open field due to favourable growing conditions in controlled environments, where temperature, humidity, and light may be regulated^11^. In controlled conditions, there is a higher total conductance to CO_2_ and stomatal conductance, resulting in better water use efficiency and increased leaf PPFD absorption. Increased stomatal conductance and total conductance to CO_2_result from optimal regimens, guaranteeing those stomata stay open for efficient gas exchange^9^. Water stress causes partial stomatal closure, which lowers transpiration and photosynthetic rates^12^. This stress negatively impacts the overall physiological performance of the chili plants by limiting CO_2_uptake, lowering assimilation rates, and decreasing the PPFD absorption by the leaves^13,14^. Higher temperatures, excessive rain, and drought are predicted effects of climate change that adversely affect the vegetative and generative growth of chili peppers^15^. Protected cultivation aim to optimize the environment while establishing a regulated microclimate that shields the crops from adverse weather, pests, and diseases^16^. Maintaining physiological homeostasis, encouraging healthy growth, and improving the quality and output of chili peppers all depend on balanced irrigation regimens customized to the plant’s growth phase^17^.

This experiment seeks to measure the physiological responses of chili peppers cultivars under various microclimates and irrigation levels, to establish reliable cultivation techniques. By systematically evaluating these factors, the study aims to offer practical suggestions for reducing the adverse effects of abiotic stress on the production of chilies, assuring resilient and sustainable methods of agriculture.

## Materials and methods

### Research location and time

This experiment was conducted at the Bale Tatanen Greenhouse, and Field Laboratory of the Faculty of Agriculture, Padjadjaran University, Jatinangor, Sumedang Regency, at an altitude of 685 m above sea level from November 2023 to March 2024.

### Research material

**Fig. 1:**
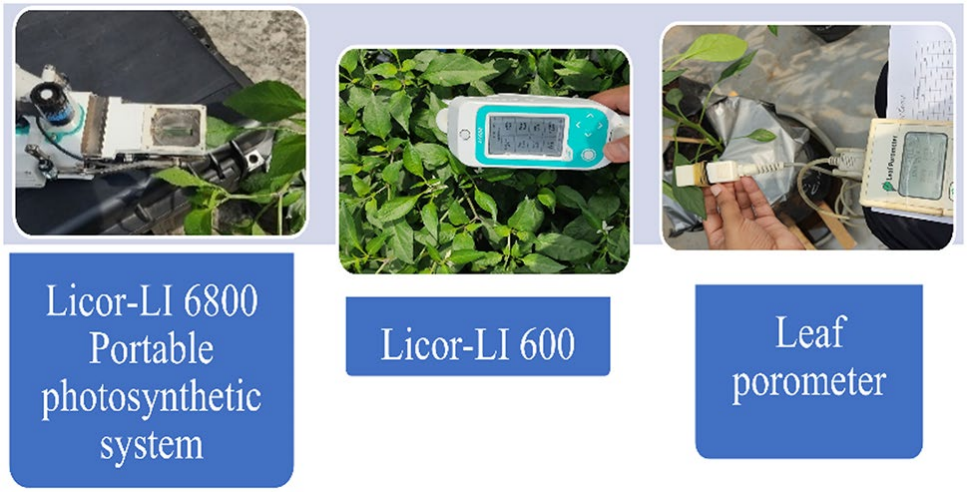
The apparatus used in the experiment for measuring the physiological parameters.

The materials used in experimental setup; mulch, scales, wrap, thermometer, hydrometer, thermohygrometer, lux meter, anemometer. The tools for physiological parameters analysis were photosynthetic analyzer LI 6800 and Li600 (Licor inc. US), Leaf porometer WTA-220 (USA) (Fig. 1). The seeds of chili pepper have been collected from university representatives upon request for the ongoing project research. As the niversity representatives take care of all these arrangements for the researches. The voucher of the specimen has been placed with them accordingly. The materials have been identified by the head of project.

### Experimental design

The experiment design using Factorial Randomized Complete Block Design was constructed by mathematical linear model equation as following:

$${\text{Y}}_{{{\text{ijk}}}} = {\text{u}} + {\text{W}}_{{\text{i}}} + {\text{C}}_{{\text{j}}} + {\text{P}}_{{\text{k}}} + \left( {{\text{WC}}} \right)_{{{\text{ij}}}} + \left( {{\text{WP}}} \right)_{{{\text{ij}}}} + \left( {{\text{CP}}} \right)_{{{\text{ij}}}} \left( {{\text{WCP}}} \right)_{{{\text{ij}}}} + {\text{r}}_{{\text{l}}} + \varepsilon _{{{\text{ijk}}}}$$

The research hypothesis:

$$\begin{aligned} & {\text{H}}_{0} :{\text{W}}_{{\text{1}}} = {\text{W}}_{{\text{2}}} = \ldots = {\text{W}}_{{\text{n}}} = 0;\;{\text{H}}_{{\text{1}}} :{\text{at}}\;{\text{least}}\;{\text{one}}\;{\text{of}}\;{\text{W}}_{{\text{i}}} \ne 0 \\ & {\text{H}}_{0} :{\text{C}}_{{\text{1}}} = {\text{C}}_{{\text{2}}} = \ldots = {\text{C}}_{{\text{n}}} = 0;\;{\text{H}}_{{\text{1}}} :{\text{at}}\;{\text{least}}\;{\text{one}}\;{\text{of}}\;{\text{C}}_{{\text{i}}} \ne 0 \\ & {\text{H}}_{0} :{\text{G}}_{{\text{1}}} = {\text{G}}_{{\text{2}}} = \ldots = {\text{G}}_{{\text{n}}} = 0;\;{\text{H}}_{{\text{1}}} :{\text{at}}\;{\text{least}}\;{\text{one}}\;{\text{of}}\;{\text{G}}_{{\text{i}}} \ne 0 \\ & {\text{H}}_{0} :\left( {{\text{WC}}} \right)_{{{\text{ij}}}} = 0\;{\text{for}}\;{\text{all}}\;{\text{i}}\;{\text{and}}\;{\text{j}};\;{\text{H}}_{{\text{1}}} :{\text{at}}\;{\text{least}}\;{\text{one}}\;{\text{of}}\;\left( {{\text{WC}}} \right)_{{{\text{ij}}}} \ne 0 \\ & {\text{H}}_{0} :\left( {{\text{WG}}} \right)_{{{\text{ij}}}} = 0\;{\text{for}}\;{\text{all}}\;{\text{i}}\;{\text{and}}\;{\text{j}};\;{\text{H}}_{{\text{1}}} :\;{\text{at}}\;{\text{least}}\;{\text{one}}\;{\text{of}}\;\left( {{\text{WG}}} \right)_{{{\text{ij}}}} \ne 0 \\ & {\text{H}}_{0} :\left( {{\text{CG}}} \right)_{{{\text{ij}}}} = 0\;{\text{for}}\;{\text{all}}\;{\text{i}}\;{\text{and}}\;{\text{j}};\;{\text{H}}_{{\text{1}}} :\;{\text{at}}\;{\text{least}}\;{\text{one}}\;{\text{of}}\;\left( {{\text{CG}}} \right)_{{{\text{ij}}}} \ne 0 \\ & {\text{H}}_{0} :\left( {{\text{WCG}}} \right)_{{{\text{ij}}}} = 0\;{\text{for}}\;{\text{all}}\;{\text{i}}\;{\text{and}}\;{\text{j}};\;{\text{H}}_{{\text{1}}} :\;{\text{at}}\;{\text{least}}\;{\text{one}}\;{\text{of}}\;\left( {{\text{WCG}}} \right)_{{{\text{ij}}}} \ne 0 \\ \end{aligned}$$

### Data analysis

Tukey Multiple Range Comparison Test is used in conjunction with post-hoc analysis to determine the optimal procedure for treatment. In contrast, LSD has the drawback of not being able to test for every combination of treatments. The Procedure of Tukey (HSD) is:

- Order treatments mean accordingly.
- Formula uses as follow:

$$~\omega = q_{ \propto } \left( {p,v} \right)\sqrt {\frac{S}{r}}$$

Where: p = treatment amount = t, v = error degree of freedom, r = replication amount, α = confident level, q_α_(p, v) = critical value that obtained from t-student table.

#### Test criteria

Compare absolute value of two different means that were distinguish the differences to HSD score.

If |µ_i_ - µ_j_| > HSD_005_, means the test result is significant.

If |µ_i_ - µ_j_| < HSD_005_, means the test result is not significant.

### Climate of the experimental setups

**Fig. 2:**
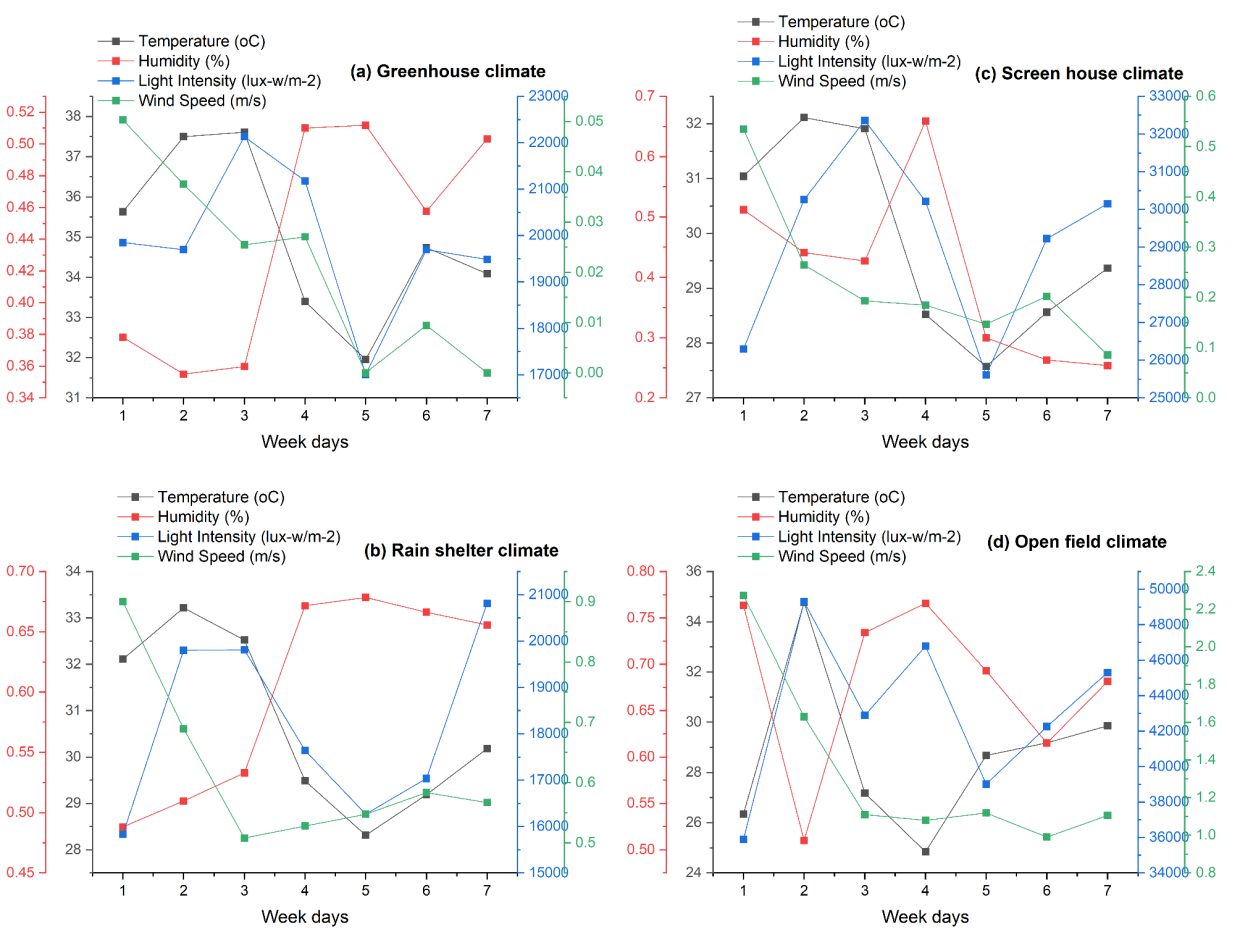
(**a**, **b**, **c**, **d**) Mean temperature, humidity, wind velocity, and light intensity in the experimental sites from the month November till March.

**Fig. 3:**
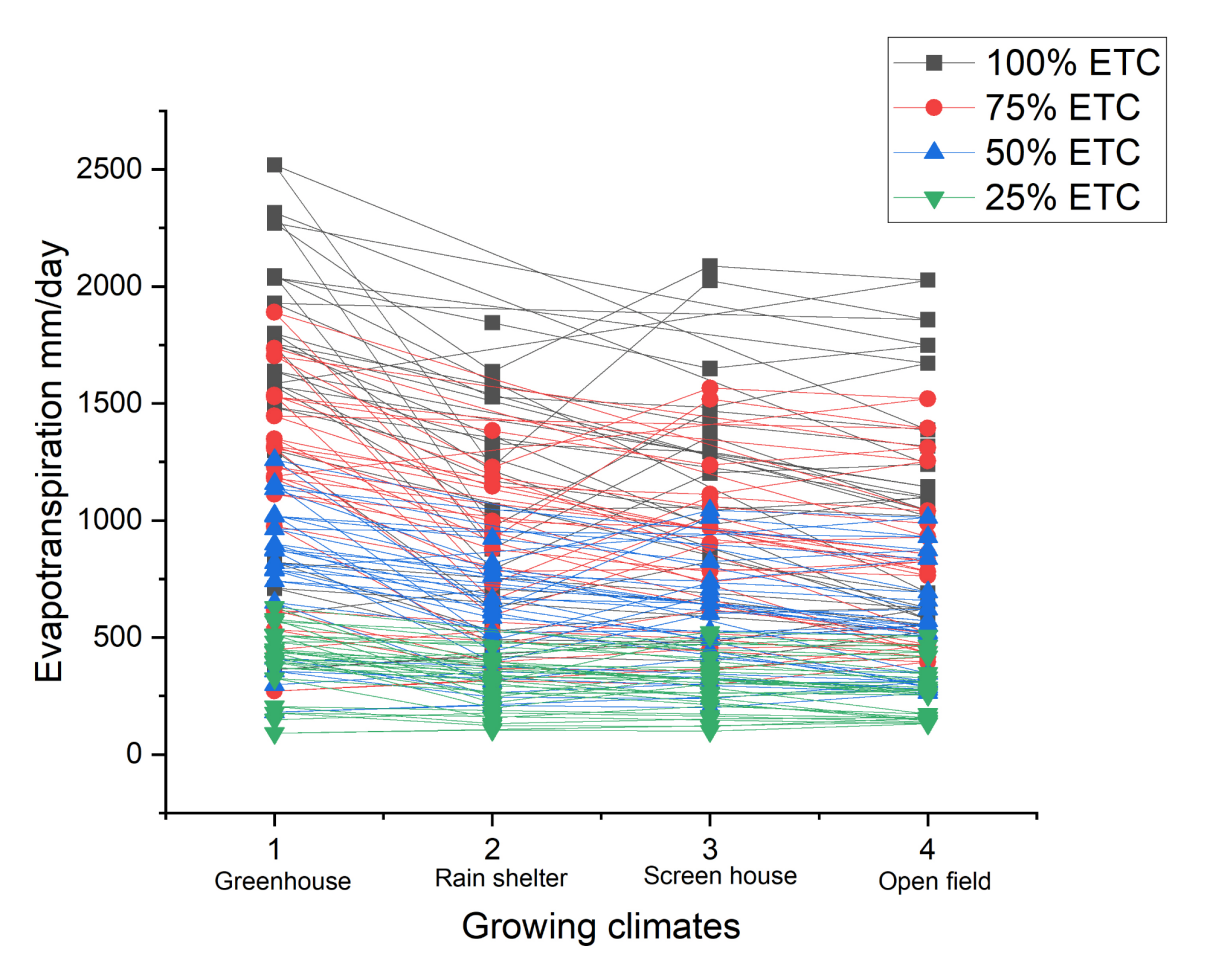
Mean evapotranspiration in each growing climates as per applied by corresponding watering applications.

#### Observations

As per observations recording the device Li-Cor 6800 was used to estimate transpiration rate (mol H_2_0 m^−2^ s^−1^), stomatal conductance to water vapour (mol m^−2^ s^−1^), Total conductance to CO_2_ (mol m^−2^ s^−1^), Photosynthetic rate (µmol m^−2^ s^−1^), Photosynthetic photon flux density (µmol m^−2^ s^−1^).

The amount of watering volume was based on plant evapotranspiration (ETc) which was calculated by the Soil Water Balance equation:

$${\text{ETc}} = {\text{P}} + {\text{I}} - {\text{R}} - {\text{D}} - \left( {{\text{W}}1 - {\text{W}}2} \right)$$

Note: ETc: Evapotranspiration (mm), P: Precipitation (mm), I: Irrigation (volume of water given) (mm), R: Run off (Surface flow) (mm), D: Drainage (Percolation) (mm), W_1_: Media weight (g) after applying water till field capacity, W_2_: Weight (g) of media on following day.

The procedure for recording day to day evapotranspiration was equipped in such a way that the three samples plant media were taken in each cultivar treatment and applied the water until field capacity and record the weight of the media and percolated water. Plant media was weighed on the following day as to note the difference of weight (evapotranspiration). The difference recorded was considered the evapotranspiration from that plant media and was assigned as 100% ETc. For 75% ETc the evapotranspiration of 100% was multiply by 0.75, for 50% multiply by 0.50 and for 25% by 0.25.

## Results

### Absolute growth rate (cm p^−1^ day^−1^)

**Fig. 4:**
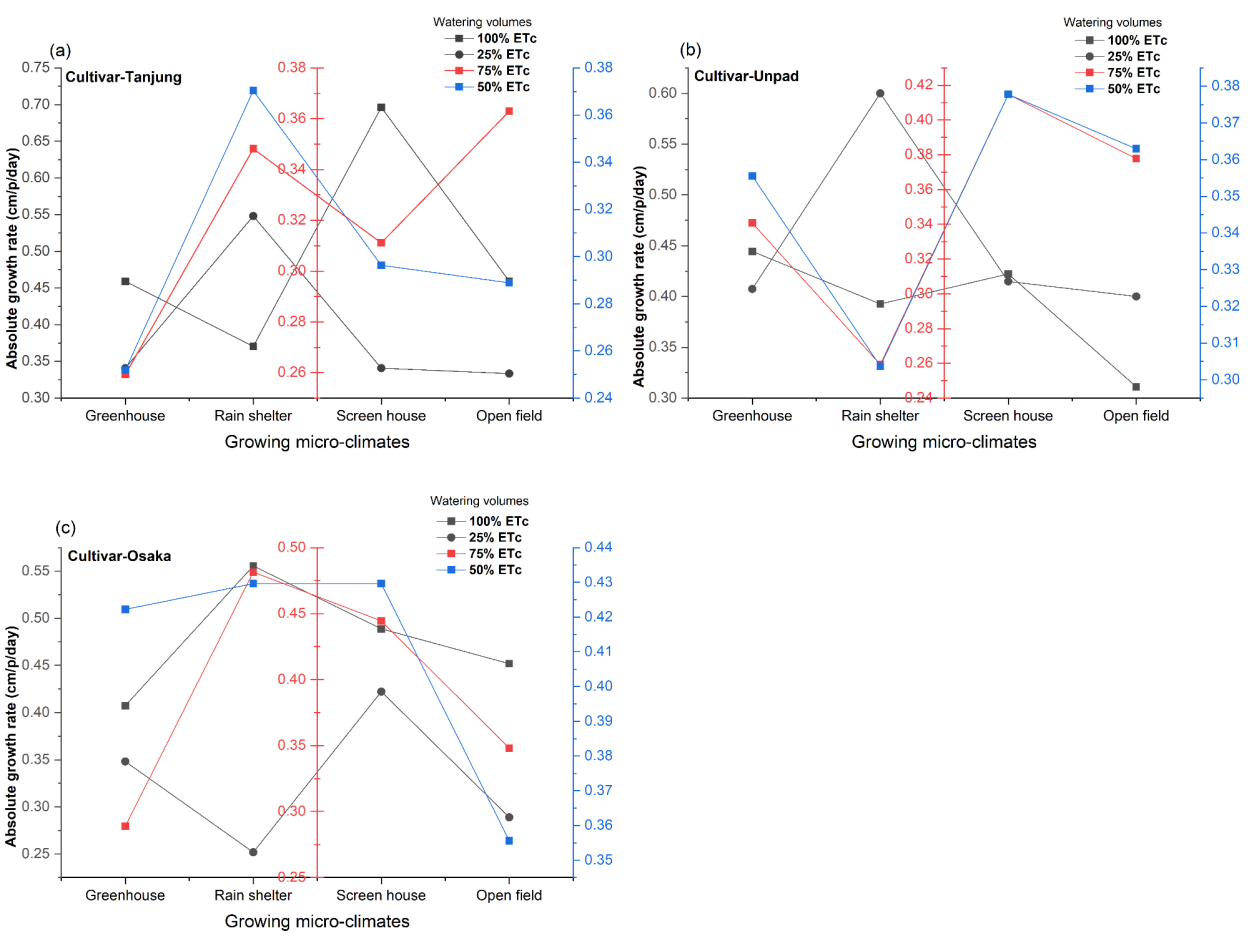
(**a**, **b**, **c**) Mean and interaction of absolute growth rate (cm p^−1^ day^−1^) in chili cultivars as affected by different growing microclimates and watering volumes.

According to statistical data analysis (Fig. 4a, b and c), cultivars have no significant (*P* > 0.05) impact on chili’s absolute growth rate (AGR), but growing microclimate conditions and watering volumes do (*P* < 0.05). The combined effects of cultivars and watering, growing climates and cultivars, and growing microclimates and watering were the only interactions that showed statistical significance (*P* < 0.05). Regarding growing microclimates, the rain shelter (0.41 cm p^−1^ day^−1^) and screen house (0.42 cm p^−1^ day^−1^) had the highest AGR values. The AGR values of 0.36 cm p^−1^ day^−1^ were lower for the greenhouse and open fields. The highest AGR value (0.45 cm p^−1^ day^−1^) for watering volumes was obtained at the 100% ETc level, followed by the 25% ETc level (0.39 cm p^−1^ day^−1^). The 50% ETc and 75% ETc levels had the lowest AGR values at 0.35 cm p^−1^ day^−1^.

### Water use efficiency (g/l)

**Fig. 5:**
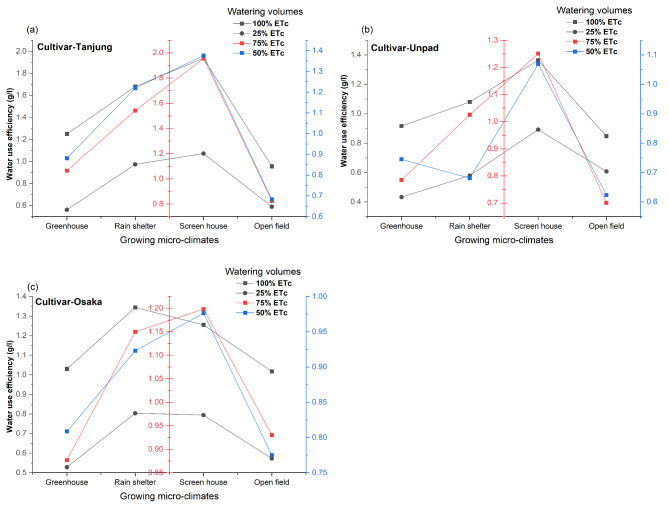
(**a**, **b**, **c**) Mean and interaction of water use efficiency (g l^−1^) in chili cultivars as affected by different growing microclimates and watering volumes.

The results of the statistical analysis (Fig. 5a, b and c) showed that, the effect of various cultivars, growing microclimates and watering volumes was determined to be significant (*P* < 0.05). Whereas, all of the interactions among the treatment were found significant (*P* < 0.05) except the three-way interaction. The screen house (1.26 g/l) and rain shelter (1.08 g/l) had the highest water use efficiency across the growing microclimates. With a value of 0.82 g/l, and 0.76 g/l the greenhouse and open field both showed reduced water use efficiency. The highest water use efficiency of 1.22 g/l was achieved at the 100% ETc level regarding watering volumes. In comparison, the efficiency of the 75% ETc, 50% ETc, and 25% ETc levels were significantly lower, at 1.10 g/l, 0.89 g/l, and 0.70 g/l, respectively. According to the study, Tanjung had the best water use efficiency (1.16 g/l) out of all the cultivars. With an efficiency of 0.93 g/l, Osaka came in second, and Unpad had the lowest efficiency of 0.84 g/l.

### Transpiration rate (mol H20 m^−2^ s^−1^)

**Fig. 6:**
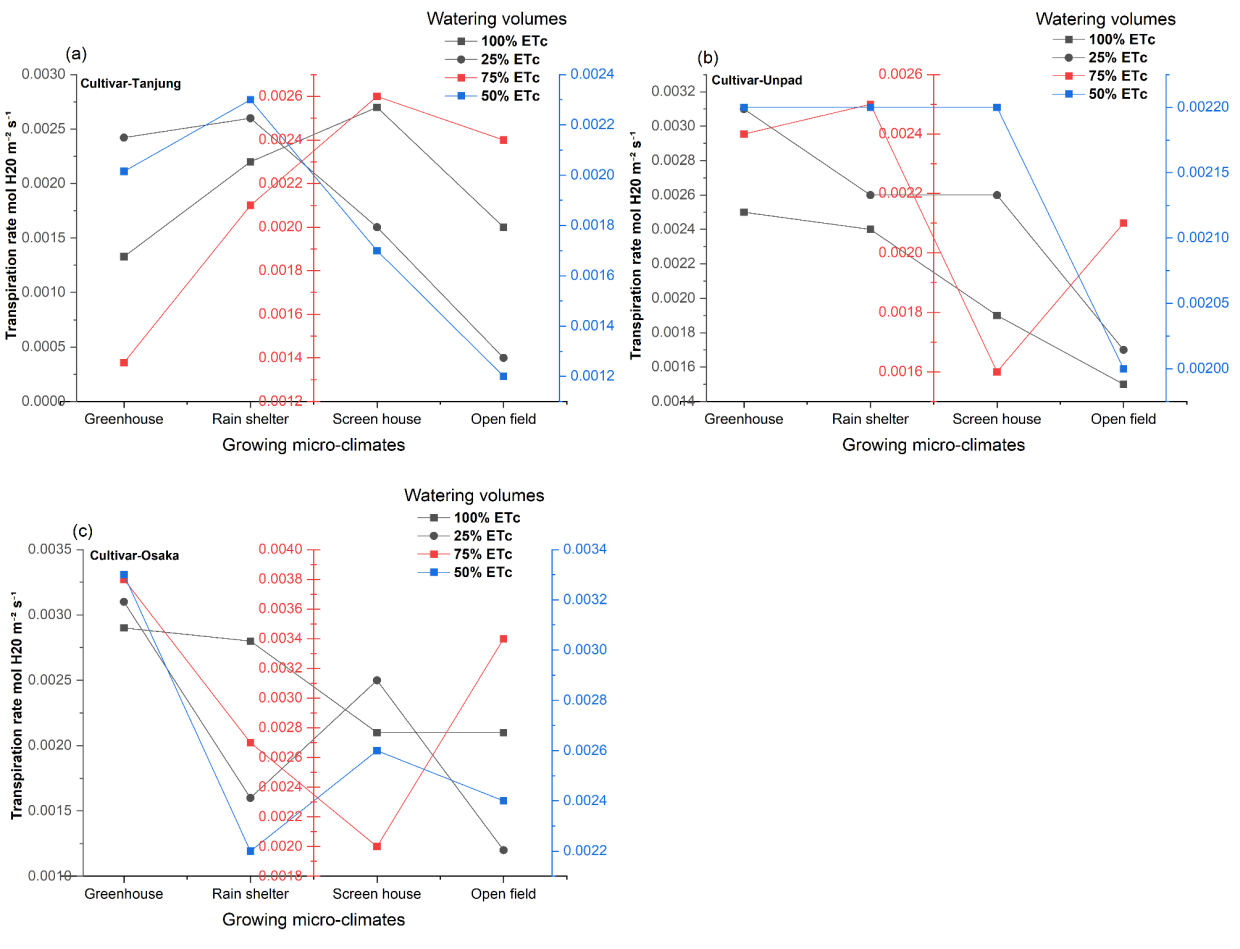
(**a**, **b**, **c**) Mean and interaction of transpiration rate (mol H_2_0 m^−2^ s^−1^) in chili cultivars as affected by different growing microclimates and watering volumes.

The statistical analysis (Fig. 6a, b and c) showed that, although the effects of various watering volumes were not statistically significant (*P* > 0.05), growing microclimate conditions and cultivars had a significant (*P* < 0.05) impact on the transpiration rate of chili. Furthermore, it was shown that none of the interactions between these treatments were significant (*P* > 0.05). The greenhouse (0.0025 mol H_2_0 m^−2^ s^−1^) and rain shelter (0.0023 mol H_2_0 m^−2^ s^−1^) had the highest transpiration rates among the growing microclimates, with the screen house (0.0021 mol H_2_0 m^−2^ s^−1^) coming in second. At 0.0018 mol H_2_0 m^−2^ s^−1^, the open field has the lowest transpiration rate. Regarding cultivars, Tanjung had a transpiration rate of 0.0019 mol H_2_0 m^−2^ s^−1^, Unpad 0.0022 mol H_2_0 m^−2^ s^−1^, and Osaka 0.0025 mol H_2_0 m^−2^ s^−1^.

### Stomatal conductance to water vapour (mol m^−2^ s^−1^)

**Fig. 7:**
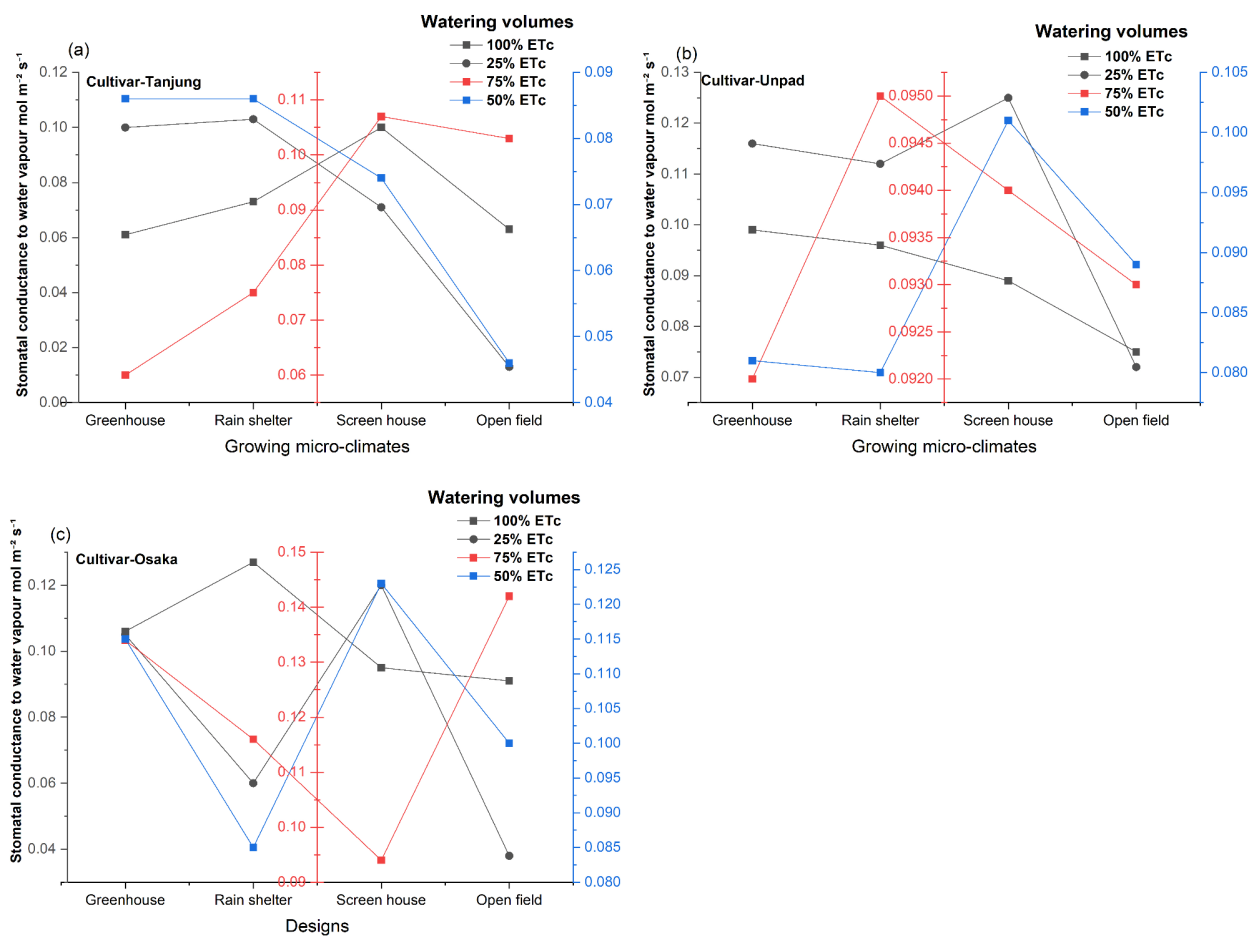
(**a**, **b**, **c**) Mean and interaction of stomatal conductance to water vapour (mol m^−2^ s^−1^) in chili cultivars as affected by different growing microclimates and watering volumes.

The statistical study results (Fig. 7a, b and c) showed that the stomatal conductance to water vapour of chili is significantly (*P* < 0.05) affected by growing microclimate conditions and cultivars but that the impact of various watering volumes was not statistically significant (*P* > 0.05). Furthermore, there was no significant (*P* > 0.05) interaction between these parameters for stomatal conductance. The screen house (0.099 mol m^−2^ s^−1^) and greenhouse (0.096 mol m^−2^ s^−1^) had the highest stomatal conductance values among the growing microclimate, with the rain shelter (0.092 mol m^−2^ s^−1^) coming in second. At 0.077 mol m^−2^ s^−1^, the open field has the lowest stomatal conductance. Regarding cultivars, Tanjung had the lowest value at 0.076 mol m^−2^ s^−1^, while Osaka and Unpad had the highest values at 0.103 mol m^−2^ s^−1^ and 0.094 mol m^−2^ s^−1^.

### Total conductance to CO2 (mol m^−2^ s^−1^)

**Fig. 8:**
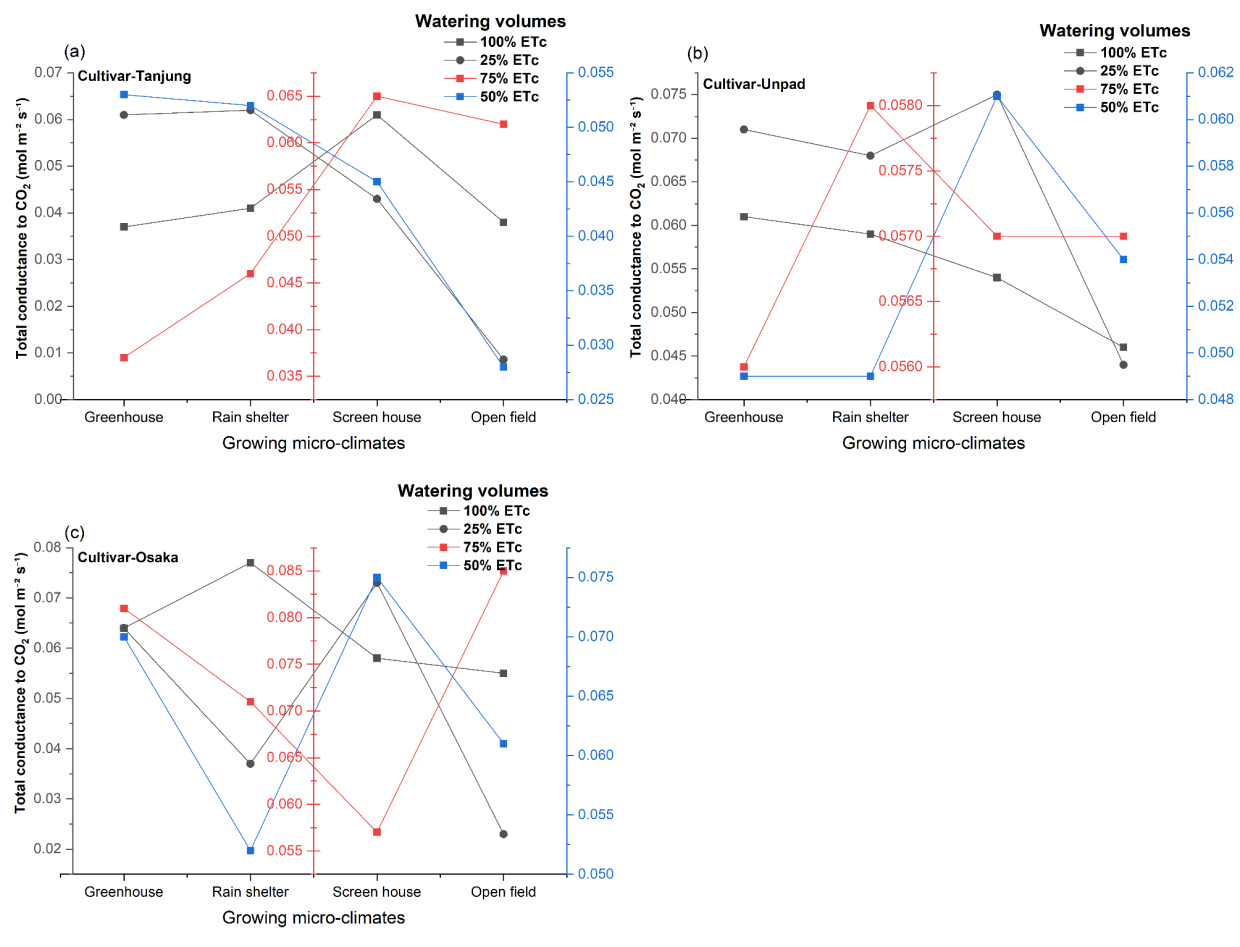
(**a**, **b**, **c**) Mean and interaction of total conductance to CO_2_ (mol m^−2^ s^−1^) in chili cultivars as affected by different growing microclimates and watering volumes.

The statistical analysis (Fig. 8a, b and c) showed that while the impact of various watering volumes was not statistically significant (*P* > 0.05), growing microclimate conditions and cultivars significantly (*P* < 0.05) affected the total conductance to CO_2_ in chili. Furthermore, it was observed that there was no significant (*P* > 0.05) interaction between any of these variables and total conductance to CO_2_. The screen house (0.060 mol m^−2^ s^−1^) and greenhouse (0.058 mol m^−2^ s^−1^) had the highest total conductance to CO_2_ values among the growing microclimate, with the rain shelter (0.056 mol m^−2^ s^−1^) coming in second. At 0.047 mol m^−2^ s^−1^, the open field has the lowest total conductance to CO_2_. Regarding cultivars, Tanjung had the lowest value at 0.046 mol m^−2^ s^−1^, while Osaka and Unpad had the highest levels at 0.062 mol m^−2^ s^−1^ and 0.057 mol m^−2^ s^−1^.

### Photosynthetic rate (µmol m^−2^ s^−1^)

**Fig. 9:**
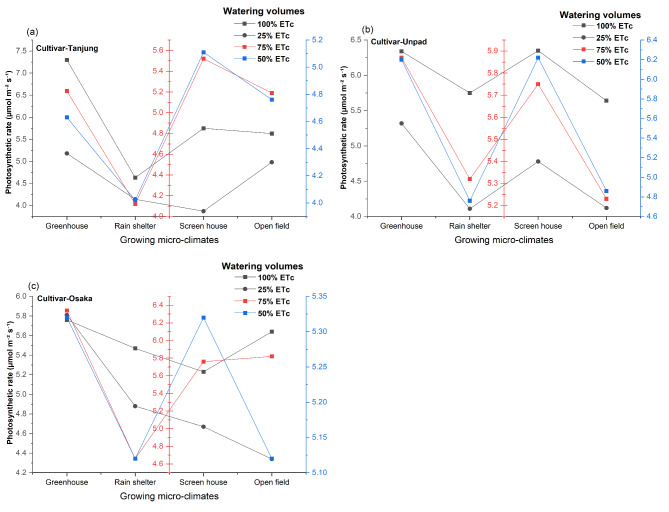
(**a**, **b**, **c**) Mean and interaction of photosynthetic rate (µmol m^−2^ s^−1^) in chili cultivars as affected by different growing microclimates and watering volumes.

According to a statistical analysis of the data (Fig. 9a, b and c), Chili’s photosynthetic rate is significantly (*P* < 0.05) affected by growing microclimates, watering volumes, and cultivars. All of these treatments’ interactions, however, were shown to be insignificant (*P* > 0.05). In terms of growing microclimates, the greenhouse had the highest photosynthetic rate (5.77 µmol m^−2^ s^−1^), followed by the screen house (5.36 µmol m^−2^ s^−1^) and open field (5.11 µmol m^−2^ s^−1^), while the rain shelter was found lower at (4.74 µmol m^−2^ s^−1^). As for cultivars, while Unpad and Osaka had higher rates at 5.41 µmol m^−2^ s^−1^ and 5.33 µmol m^−2^ s^−1^, respectively, Tanjung had a lower rate at 5.01 µmol m^−2^ s^−1^. When it came to watering volumes applications, the photosynthetic rate at the 100% ETc level was the highest at 5.79 µmol m^−2^ s^−1^, followed by levels at 75% ETc (5.39 µmol m^−2^ s^−1^) and 50% ETc (5.12 µmol m^−2^ s^−1^) and the lowest at 4.68 µmol m^−2^ s^−1^ at the 25% ETc level.

### Photosynthetic photon flux density (µmol m^−2^ s^−1^)

**Fig. 10:**
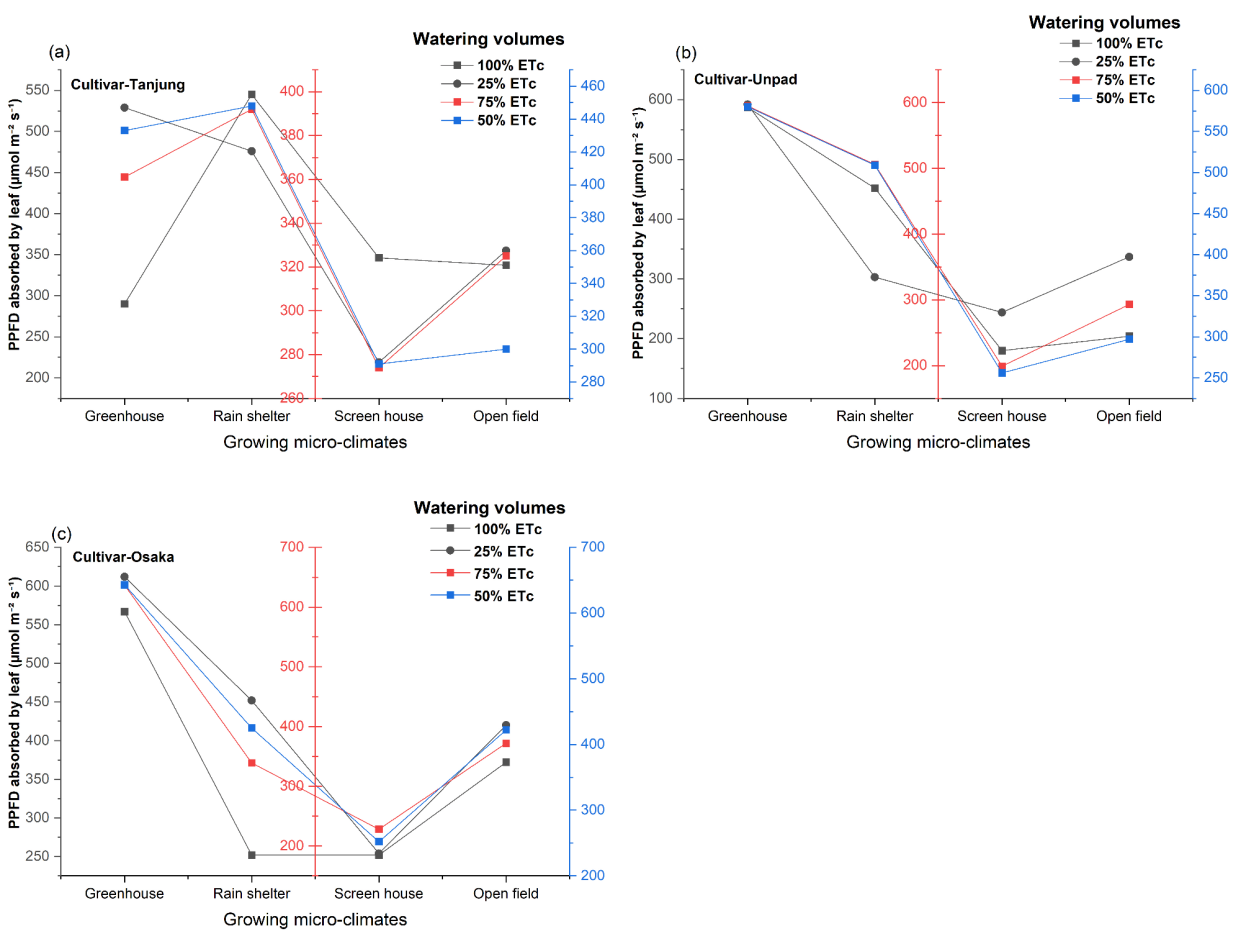
(**a**, **b**, **c**) Mean and interaction of photosynthetic photon flux density absorbed by leaf (µmol m^−2^ s^−1^) in chili cultivars as affected by different growing microclimates and watering volumes.

The results of the statistical analysis of the data (Fig. 10a, b and c) showed that, in contrast to cultivars and watering volumes, growing microclimates significantly (*P* < 0.05) affect the Photosynthetically Photon Flux Density (PPFD) of chili. The interaction between cultivars and growing microclimates was the only treatment interaction that showed statistical significance (*P* < 0.05), whereas the other interactions among the treatments were found to be non-significant (*P* > 0.05). The greenhouse (535.5 µmol m^−2^ s^−1^) had the greatest PPFD value among the growing climates, followed by the rain shelter (424.9 µmol m^−2^ s^−1^). The PPFD values of the open field and screen house were lower, at 336.1 µmol m^−2^ s^−1^ and 249.6 µmol m^−2^ s^−1^.

### Pearson correlation

**Fig. 11:**
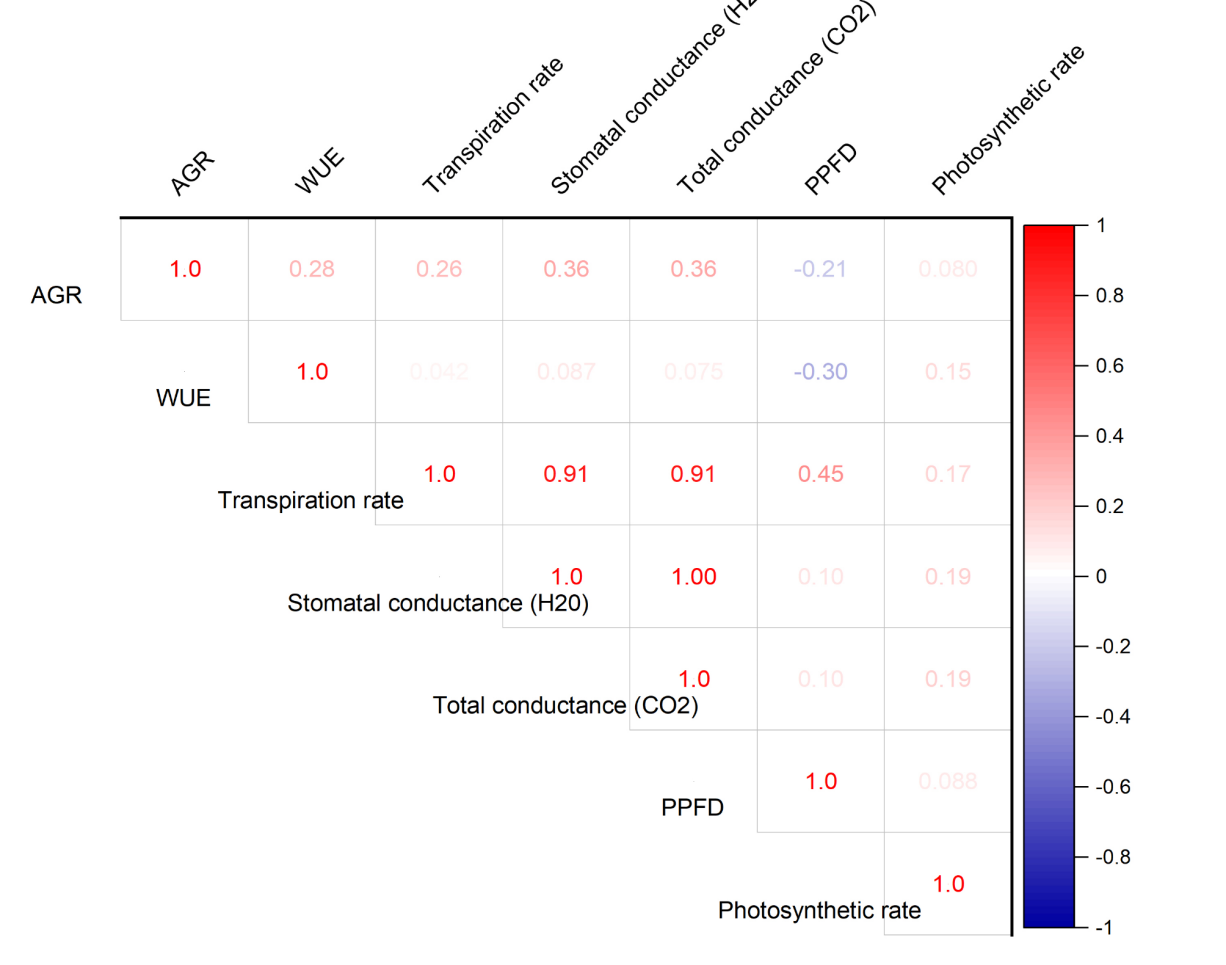
Pearson correlation matrix illustrating the relationships between major environmental and physiological parameters in chili plant.

**Fig. 12:**
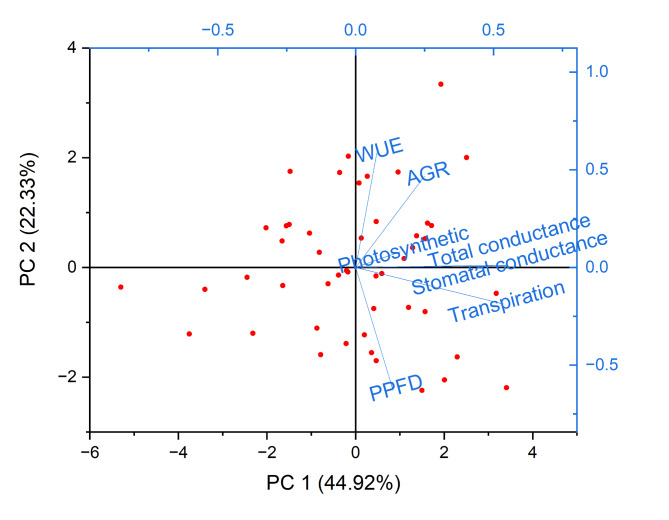
This graph, a biplot from a Principal Component Analysis (PCA), shows how several physiological variables relate. The y-axis (PC2) accounts for 22.33% of the variation, and the x-axis (PC1) for 44.92%. The close clustering of variables like photosynthetic rate, stomatal conductance, transpiration, and PPFD (photosynthetic photon flux density) suggests a positive correlation between them. The observation that AGR (Absolute Growth Rate) and WUE (Water Use Efficiency) are slightly apart indicates that their application patterns differ from those of the other variables on these significant components.

The correlations between several physiological and environmental factors that are important to plant growth are shown in this correlation matrix (Fig. 11). The correlations between transpiration rate, stomatal conductance, and total conductance to CO_2_ are all highly positive (all at 0.91 and above). It indicates that the others tend to increase with the increase in one of these components. The AGR (cm/p/day) and stomatal conductance (0.36), as well as total conductance to CO_2_ (0.36), show a slightly positive correlation, indicating that higher conductance is linked to faster growth rates. A weak correlation exists between higher AGR and lower PPFD levels, though this correlation is insignificant. AGR and PPFD have a weak negative correlation (−0.21). Most other relationships exhibit weak correlations, indicating little to no linear correlation, such as WUE with different parameters (Fig. 12).

## Discussion

The variability among cultivars may be obscured by the more pronounced impacts of environmental factors. The results can be attributed to the microclimate’s-controlled environments, which lessen stressors like wind, severe heat, and direct sunlight^4^ (Fig. 2). Regulated environments preserve humidity and temperature at appropriate levels, conditions of utmost importance for the physiological processes that control ideal plant growth^18^. Potential factors like high humidity and inadequate temperature control may cause the greenhouse’s slower growth rates^5^ as such conditions can be more challenging for plants to transpire and absorb nutrients. The plants were probably subjected to stressors like severe temperatures, pests, and diseases in the open field, where they were vulnerable to the full brunt of environmental changes^19,20^ (Fig. 2). The variations in temperature, humidity, and air movement within microclimates can be attributed as causes for the variations in transpiration rates^2^. Microclimate significantly affect stomatal conductance, which aligns with previous research highlighting the impact of environmental constraints on plant physiology^21,22^. Mitigating severe temperatures and reducing water deficit, these structures provide a steady and controlled environment that fosters appropriate stomatal function^23^. The severe environmental factors in open fields, such as higher temperatures, wind exposure, and evolving humidity levels, can cause increased water stress and stomatal closure^24^. Reduced stomatal conductance can result from a decrease in the relative water content of leaves, which can lower the amount of water that plants utilize overall^9^. Protected environments provide optimal light, temperature, and humidity conditions, improving photosynthetic activity^25^. The screen house minimizes stress factors impeding photosynthetic efficiency by providing an appropriate ratio between protection and environmental exposure^26^. The greenhouse’s-controlled conditions, maximize light penetration and minimize external environmental stressors such as strong wind and precipitation, which can restrict light availability, are responsible for the greater PPFD^27^. A lower and less constant PPFD results from the open field’s exposure to full environmental variations, which cause unpredictability in light availability due to factors like cloud cover and shading from nearby plants^28^.

A sufficient water supply ensures the plants can support nutrient intake, maintain turgor pressure, and continue metabolic processes vital for growth^29^. The amounts of water deficit were insufficient to activate adaptive systems, they were significant enough to disrupt typical physiological processes^13^. The optimal watering schedule best maximizes WUE, and the right amount of irrigation improves resource use. Controlled conditions that reduce water loss through evapotranspiration (Fig. 3) are responsible for the high WUE observed in such microclimates^17^. Higher temperatures can increase transpiration rates, due to a potential increase in VPD, leaf temperature and cuticular water permeability^3^. Increased environmental stresses like wind, light intensity, and higher temperatures cause stomatal closure to preserve water, accounting for the lowest open field transpiration rate^14^. Chili plants have adaptation mechanisms that protect stomatal conductance from variations in water availability^30^. The photosynthetic machinery is assisted by adequate water availability, which ensures effective gas exchange and nutrient transport^8^. At 60% water availability, water use efficiency rises; however, at 80% and 100% water availability, it declines^7^.

The variation in transpiration rates among the cultivars signifies genetic variations in how they relate to water usage. The cultivar may have a more active water uptake strategy, as suggested by its increased transpiration rate, which supports the theory of genotype-specific environmental responses^10^. The cultivars generally sustain higher gas exchange rates and photosynthesis, which can be desirable in conditions where water is easily accessible but could be detrimental in drought-prone regions^31^. The difference in stomatal conductance between cultivars emphasizes the importance of carefully choosing plant kinds, considering the availability of water and particular environmental factors^4^. Masabni et al.^32^ highlighted how genetic traits influence how plants react to environmental stress. Cultivars with higher photosynthetic rates have genetic traits that enhance their capacity to transform light into chemical energy efficiently^33^. Increased photosynthetic efficiency, improved source-to-sink relationship within the plant, decreased respiration, increased translocation, and sugar and other metabolite accumulation^34^.

## Conclusion

This study reveals essential physiological and growth responses under various circumstances, highlighting the complex interactions between cultivars of chili peppers, watering volumes, and growing microclimates. Despite some genetic variations between cultivars, microclimate factors and water availability have a more significant influence on growth and physiological metrics like absolute growth rate (AGR), water use efficiency (WUE), transpiration rate, stomatal conductance, photosynthetic rate. Higher AGR and WUE values resulted from optimal growth being assisted by the controlled settings of the rain shelter and screen house. Significantly, the maximum growth and physiological performance were consistently obtained with 100% ETc watering volumes, highlighting the vital importance of a sufficient water supply for preserving plant health and optimizing output. Tanjung usually performs higher in terms of WUE and lower in stomatal conductance, highlighting the differences in responses among cultivars and emphasizing the significance of choosing suitable genotypes for certain environmental circumstances and resource availability. These results suggest that controlled settings and well-designed watering schedules should be strategically used to reduce the adverse effects of abiotic stress and increase the resilience and sustainability of chili production. In light of ongoing climate change, this study offers insightful guidance for creating realistic and robust cultivation systems that can significantly support sustainable agriculture practices.

## Data Availability

All of the research data are presented in this manuscript, however about the raw data it will be shared upon the request to authors of this article. The main author Farhan Ahmad should be contacted for sharing the raw data. Farhan Ahmadfarhan22018@mail.unpad.ac.id.

## References

[1] Rajametov, S. N., Yang, E. Y. & Cho, M. C. Heat-tolerant hot pepper exhibits constant photosynthesis via increased transpiration rate, high proline content and fast recovery in heat stress condition. *Sci. Rep.*(0123456789), 1–9. 10.1038/s41598-021-93697-5 (2021). 10.1038/s41598-021-93697-5 PMC8275607 34253784

[2] Widuri, L. I. et al. Short-term drought exposure decelerated growth and photosynthetic activities in Chili pepper (Capsicum annuum L). *Ann. Agric. Sci.* **65**(2), 149–158. 10.1016/j.aoas.2020.09.002 (2020).

[3] Banjare, C., Mahanta, D. & Sahu, P. A comprehensive review on protected cultivation: Importance, scope and status. **14**(7), 46–55 (2024).

[4] Ouzounidou, G. et al. Comparative study on the effects of various plant growth regulators on growth, quality and physiology of Capsicum annuum L. **42**(2), 805–814 (2010).

[5] Buendia-Valverde, M. et al. Effects of cadmium, thallium, and vanadium on photosynthetic parameters of three chili pepper (Capsicum annuum L.) varieties. 1–14 (2023). 10.3390/plants12203563 PMC10609808 37896025

[6] Jain, S. et al. A Comprehensive review on protected cultivation of horticultural crops: Present status and future prospects. *Int. J. Environ. Clim. Chang.* **13**(11), 3521–3531. 10.9734/ijecc/2023/v13i113528 (2023).

[7] Sinaga, R. Physiological response of three varieties of cayenne pepper (Capsicum Frutescens) to decreased water availability. *Int. J. Ecophysiol.* **2**(02), 129–136. 10.32734/ijoep.v2i02.4684 (2020).

[8] Putra, G. M. D., Sutiarso, L., Nugroho, A. P., Ngadisih & Chaer, M. S. I. Application of machine learning to study effect of environmental manipulation in frame of smart agriculture on the stomata of Capsicum annuum. *IOP Conf. Ser. Earth Environ. Sci.* **1059**(1), 0–13. 10.1088/1755-1315/1059/1/012034 (2022).

[9] Guang-Cheng, S., Rui-Qi, G., Na, L., Shuang-En, Y. & Weng-Gang, X. Photosynthetic, chlorophyll fluorescence and growth changes in hot pepper under deficit irrigation and partial root zone drying. *Afr. J. Agric. Res.* **6**, 4671–4679. 10.5897/AJAR11.812 (2011).

[10] Wen, Y., Zhang, Y., Cheng, R. & Li, T. Photosynthetic induction of the leaves varies among pepper cultivars due to stomatal oscillation. *Sci. Hortic. (Amsterdam)*. **318**, 112126. 10.1016/j.scienta.2023.112126 (2023).

[11] Abdel-Galil, H. S. Performance of a poly-greenhouse with different ventilation gaps under Fayoum climatic conditions. *Misr J. Agric. Eng.* **28**(4), 1197–1214. 10.21608/mjae.2011.102637 (2011).

[12] Sharma, H., Shukla, M. K., Bosland, P. W. & Steiner, R. Soil moisture sensor calibration, actual evapotranspiration, and crop coefficients for drip irrigated greenhouse Chile peppers. *Agric. Water Manag.* **179**, 81–91. 10.1016/j.agwat.2016.07.001 (2017).

[13] Sharma, H., Shukla, M. K., Bosland, P. W. & Steiner, R. L. Physiological responses of greenhouse-grown drip-irrigated chile pepper under partial root zone drying. *HortScience* **50**(8), 1224–1229. 10.21273/hortsci.50.8.1224 (2015).

[14] Kabir, M. Y., Nambeesan, S. U., Bautista, J. & Díaz-Pérez, J. C. Effect of irrigation level on plant growth, physiology and fruit yield and quality in bell pepper (Capsicum annuum L.). *Sci. Hortic. (Amsterdam).* **281**(December 2021). 10.1016/j.scienta.2021.109902 (2020).

[15] Khaitov, B. et al. Importance and production of chilli pepper; heat tolerance and efficient nutrient use under climate change conditions. *Korean J. Agric. Sci.* **46**(4), 769–779. 10.7744/kjoas.20190059 (2019).

[16] Gruda, N. & Tanny, J. *Horticulture: Plants for people and places*, vol. 1. 10.1007/978-94-017-8578-5 (2013).

[17] Duah, S. A. et al. Effect of water supply on physiological response and phytonutrient composition of Chili peppers. *Water (Switzerland)* **13**(9). 10.3390/w13091284 (2021).

[18] Palma, M., Sevilla, F., Jime, A., Rı, L. A. & Corpas, F. J. Physiology of pepper fruit and the metabolism of antioxidants: Chloroplasts, mitochondria and peroxisomes. 627–636. 10.1093/aob/mcv121 (2015). 10.1093/aob/mcv121 PMC4578004 26220658

[19] Park, B. M. et al. Differential responses of cherry tomatoes (Solanum lycopersicum) to long-term heat stress. *Horticulturae* **9**(3), 1–13. 10.3390/horticulturae9030343 (2023).

[20] Nawaz, H., Akgün, İ. & Şenyiğit, U. Effect of deficit irrigation combined with Bacillus simplex on water use efficiency and growth parameters of maize during vegetative stage. *BMC Plant. Biol.* **24**(1), 1–11. 10.1186/s12870-024-04772-8 (2024). 38403579 10.1186/s12870-024-04772-8 PMC10895846

[21] Zamljen, T., Zupanc, V. & Slatnar, A. Influence of irrigation on yield and primary and secondary metabolites in two chilies species, Capsicum annuum L. and Capsicum chinense Jacq. *Agric. Water Manag.* **234**, 106104. 10.1016/j.agwat.2020.106104 (2020).

[22] Iqbal, B. et al. Advancing environmental sustainability through microbial reprogramming in growth improvement, stress alleviation, and phytoremediation. *Plant. Stress* **10**, 100283. 10.1016/j.stress.2023.100283 (2023).

[23] Siaga, E. et al. Growth and morpho-physiological assessments of Indonesian red chili cultivars on early vegetative stage under water stress conditions: A comparison of waterlogging and drought. *Chil. J. Agric. Res.* **84**(3), 425–438. 10.4067/S0718-58392024000300425 (2024).

[24] Nazir, M. J. et al. Harnessing soil carbon sequestration to address climate change challenges in agriculture. *Soil. Tillage Res.* **237**, 105959. 10.1016/j.still.2023.105959 (2024).

[25] Magaña-López, E. et al. Nanostructured mesoporous silica materials induce hormesis on chili pepper (Capsicum annuum L.) under greenhouse conditions. *Heliyon* **8**(3). 10.1016/j.heliyon.2022.e09049 (2022). 10.1016/j.heliyon.2022.e09049 PMC8917290 35287323

[26] Salas, R. A. Effects of physical barrier and insect growth regulator on whitefly control and yield of chili pepper (Capsicum annuum L). *J. Food Nutr. Sci.* **3**(1), 13. 10.11648/j.jfns.s.2015030102.13 (2015).

[27] Aladenola, O. & Madramootoo, C. Response of greenhouse-grown bell pepper (Capsicum annuum L.) to variable irrigation. *Can. J. Plant. Sci.* **94**(2), 303–310. 10.4141/CJPS2013-048 (2014).

[28] Zhang, D. et al. A comparative overview on chili pepper (Capsicum genus) and sichuan pepper (zanthoxylum genus): From pungent spices to pharma-foods. *Trends Food Sci. Technol.* **117**(October 2020), 148–162. 10.1016/j.tifs.2021.03.004 (2020).

[29] Fernández, M. D. et al. Water use and production of a greenhouse pepper crop under optimum and limited water supply. *J. Hortic. Sci. Biotechnol.* **80**(1), 87–96. 10.1080/14620316.2005.11511897 (2005).

[30] Estrada-Luna, A. A., Davies, J. T. & Egilla, J. N. Physiological changes and growth of micropropagated chile ancho pepper plantlets during acclimatization and post-acclimatization. *Plant. Cell. Tissue Organ. Cult.* **66**(1), 17–24. 10.1023/A:1010606430060 (2001).

[31] Hussein, M. M., El-Faham, S. Y. & Alva, A. K. Pepper plants growth, yield, photosynthetic pigments, and total phenols as affected by foliar application of potassium under different salinity irrigation water. *Agric. Sci.* **03**(02), 241–248. 10.4236/as.2012.32028 (2012).

[32] Masabni, J., Sun, Y., Niu, G. & Valle, P. D. Shade effect on growth and productivity of tomato and chili pepper. *Horttechnology* **26**(3), 344–350. 10.21273/horttech.26.3.344 (2016).

[33] Medrano, H., Escalona, J. M., Bota, J., Gulías, J. & Flexas, J. Regulation of photosynthesis of C3 plants in response to progressive drought: Stomatal conductance as a reference parameter. *Ann. Bot.* **89**, 895–905. 10.1093/aob/mcf079 (2002). 12102515 10.1093/aob/mcf079 PMC4233802

[34] Zakaria, N. I., Ismail, M. R., Awang, Y., Megat Wahab, P. E. & Berahim, Z. Effect of root restriction on the growth, photosynthesis rate, and source and sink relationship of chilli (Capsicum annuum L.) grown in soilless culture. *Biomed Res. Int.* **2020**. 10.1155/2020/2706937 (2020). 10.1155/2020/2706937 PMC7008264 32090071

